# Decellularized Extracellular Matrix-Based Bioinks for Tendon Regeneration in Three-Dimensional Bioprinting

**DOI:** 10.3390/ijms232112930

**Published:** 2022-10-26

**Authors:** Fouad Al-Hakim Khalak, Fátima García-Villén, Sandra Ruiz-Alonso, José Luis Pedraz, Laura Saenz-del-Burgo

**Affiliations:** 1 NanoBioCel Group, Laboratory of Pharmaceutics, School of Pharmacy, University of the Basque Country (UPV/EHU), 01006 Vitoria-Gasteiz, Spain; 2 Biomedical Research Networking Center in Bioengineering, Biomaterials and Nanomedicine (CIBER-BBN), Health Institute Carlos III, Monforte de Lemos 3-5, 28029 Madrid, Spain; 3 Bioaraba Health Research Institute, Jose Atxotegi, s/n, 01009 Vitoria-Gasteiz, Spain

**Keywords:** tissue engineering, decellularized extracellular matrix, 3D bioprinting, tendon

## Abstract

In the last few years, attempts to improve the regeneration of damaged tendons have been rising due to the growing demand. However, current treatments to restore the original performance of the tissue focus on the usage of grafts; although, actual grafts are deficient because they often cannot provide enough support for tissue regeneration, leading to additional complications. The beneficial effect of combining 3D bioprinting and dECM as a novel bioink biomaterial has recently been described. Tendon dECMs have been obtained by using either chemical, biological, or/and physical treatments. Although decellularization protocols are not yet standardized, recently, different protocols have been published. New therapeutic approaches embrace the use of dECM in bioinks for 3D bioprinting, as it has shown promising results in mimicking the composition and the structure of the tissue. However, major obstacles include the poor structural integrity and slow gelation properties of dECM bioinks. Moreover, printing parameters such as speed and temperature have to be optimized for each dECM bioink. Here, we show that dECM bioink for 3D bioprinting provides a promising approach for tendon regeneration for future clinical applications.

## 1. Introduction

The musculoskeletal (MSK) system is composed of tendons, ligaments, bones, and muscles, whose main function is to enable locomotion. The forces that originate in the muscles are transferred through the tendons and the ligaments to the bones [1]. Severe injuries, such as ruptures and tears in the musculoskeletal system, often require complex surgeries utilizing grafts that have been obtained from different biological sources [2]. More than one third of all of the known musculoskeletal medical cases are made up of tendon and ligament injuries, with approximately four million new cases worldwide every year. Tendon injuries alone are prevalent worldwide, with 30 million surgical procedures being performed annually. The rotator cuff (RC) tendon is one of the most common injury sites leading to shoulder dysfunction and recurrent pain [3,4,5,6]. Anatomic failure of RC repairs has been shown to be 27% after 23 months [1].

The procedure to repair tendon lesions often includes suturing and reattaching or the use of grafts. However, the lack of cells and supply of blood vessels often end in retearing or further complications, such as abnormal ossification and post-surgical adhesion. Although grafts are preferred for large tendon ruptures because of their good mechanical strength and their ability to promote cell proliferation and tissue growth, they have many disadvantages. These include donor site morbidity, low availability, inflammatory response, and infection risk [2,5,7]. Presently, autografts are the preferred method for tissue replacements, and while allografts and xenografts represent viable alternatives, they have some transplantation problems, such as immune incompatibility, the transmission of infectious diseases, and the risk of rejection. As an alternative, newer and more efficient tissue engineering techniques are being researched [1,8].

Decellularized tendon scaffolds have great potential for tendon repair [9]. They have been shown to maintain the tissue biomechanical properties and composition, including tendon-specific structural proteins, growth factors, and cytokines [9,10]. However, for their clinical application, decellularized tendon scaffolds still have many drawbacks, such as cell source, ex vivo regeneration [11], limited shapes, and donor shortage. In order to overcome these complications, decellularized tendons are used as a novel biomaterial in 3D bioprinting as another tissue engineering approach [12,13].

**Figure 1:**
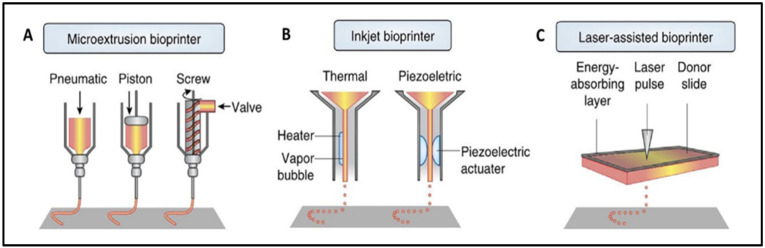
Visual representation of extrusion (**A**), inkjet (**B**), and laser-assisted (**C**) bioprinters. Extrusion bioprinter (**A**) can have pneumatic, piston, or screw-driven pressure. Inkjet bioprinter (**B**) can be thermal or piezoelectric. Reproduced with permission [15].

There are numerous techniques for manufacturing scaffolds, with 3D bioprinting being the most widely used. Three-dimensional bioprinting uses computer-designed 3D models and bioinks to fabricate scaffolds with specific and complex structures by using the layer-by-layer method, which mimics the native extracellular matrix (ECM) structure [3,14]. Custom designs can be made from a clinical image of the lesion in order to repair the patient’s defect [1]. The most frequently reported bioprinting technologies are extrusion (76%), droplet (14%), and laser-assisted bioprinting (8%), as shown in Figure 1 [15].

The extrusion technique relies on applying pressure (pneumatic, piston-driven, screw-driven) to a container of bioink in order to push it out of the printhead. It deposits uninterrupted filaments of bioink [1]. These types of bioprinters can have coaxial nozzles or multi-printheads to create vascular structures or multi-tissue scaffolds [14]. Among its advantages are its low printing cost, its ease to use, its capacity to sustain a high cellular density, and its ability to print different biomaterials [1,8,14,16,17]. However, it has a low print resolution, and the cell viability is affected when the print pressure and the shear force increase [1,14,18].

Inkjet bioprinting works by generating thermal or piezoelectric forces that release droplets of bioink from the printhead. It is a low-cost technique with a high printing accuracy and a fast molding speed. It requires low viscosity bioinks, limiting the structures that can be bioprinted and the cell density of the bioink. Other limitations include the variability in droplet size and nozzle clogging [1,14].

For laser-assisted bioprinting, a ribbon, an absorbing layer that is normally coated with gold or titanium, and the bioink is used. The laser pulse triggers the release of the bioink droplets. Typically, the cell viability is high, but it depends on the parameters of laser’s printing process. It is a high-resolution technique, but its biggest disadvantage is its high price [1,19].

In 3D bioprinting, the selection of the bioinks’ composition is a crucial step [20]. The bioinks allow for the addition of the different cell types, biomaterials, and growth factors that are utilized in clinical therapies to simulate the structure of different tissues [5,21]. Biomaterials play a vital role in the regeneration of tissues, which gives structural support and adequate biomechanical properties that simulate the original microenvironment [4]. Biomaterials are classified as natural or synthetic materials depending on their source. Natural biomaterials, such as collagen, hyaluronic acid, and chondroitin sulfate, are extracted from natural sources of ECM. These materials create suitable microenvironments in order to support essential cell interaction (proliferation, adhesion, and differentiation) [8,22]. However, natural biomaterials are difficult to handle, and their mechanical strength is poor. Therefore, their use in tissues that support great load-bearing is limited [13,23]. In order to improve the mechanical properties, synthetic-based polymers are used [1,10]. Synthetic polymers are easier to manipulate and they enhance the bioink properties, but they do not have good biocompatibility properties due to the lack of any cell proliferation cue. Hence, it is common to combine different types of materials in order to acquire their best properties [23].

Nevertheless, an inflammatory response and an immune rejection can be triggered by the existence of allogenic and xenogenic antigens in the transplanted biomaterials. Other significant drawbacks of scaffold production are the heterogeneous distribution of cells, which may result in a dysfunctional construct and the tissues’ lack of vascularization [8,22].

**Figure 2:**
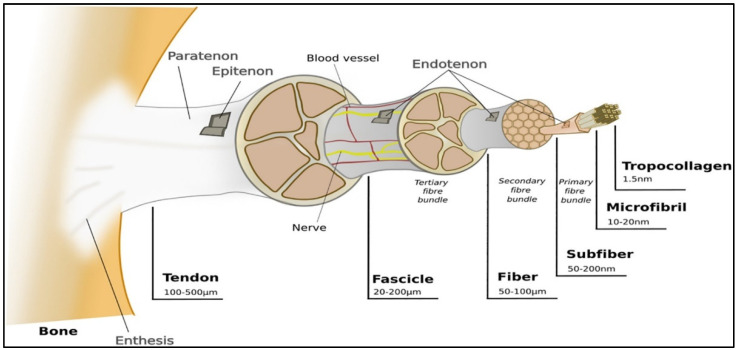
Representation of ligaments hierarchical structure. Reproduced with permission [3].

So far, bioprinted constructs have not been shown to mimic the ECM microenvironment [19]. The ECM is the native 3D microenvironment of local cells. It is a vital and complex meshwork where structural and functional proteins, such as collagens (e.g., Collagen I), glycosaminoglycans (GAGs) (e.g., heparin and chondroitin sulfate), proteoglycans (e.g., decorin), and adhesion molecules (e.g., fibronectin), can be found. For any given tissue type, the ECM has an appropriate architectural design where there is a variation in the orientation, the nature, and the number of matrix components among the different tissues [24,25,26]. Tendons are mainly composed of ECM, where 68% corresponds to water, 30% to collagen, and 2% to elastin. Unlike cartilage tissue, the main collagen found is type I (95%). Tendons have characteristics from the unidirectional orientation of collagen type I molecules and tenocytes [8]. Collagen fibers are set into larger progressive subunits, as shown in Figure 2.

Mimicking the 3D structure of ECM is complicated. In recent advances in bioinks, decellularized ECM (dECM) has been considered to be a novel biomaterial for 3D bioprinting [21,22]. The bioinks containing this biomaterial are known to possess a higher regenerative capacity than those including alginate, collagen, hyaluronic acid, and fibrin, among others [21]. The presence of the dECM in the bioink increases cell differentiation and proliferation because it expresses tissue-specific cues such as differentiation markers and proteins [19,27].

**Figure 3:**
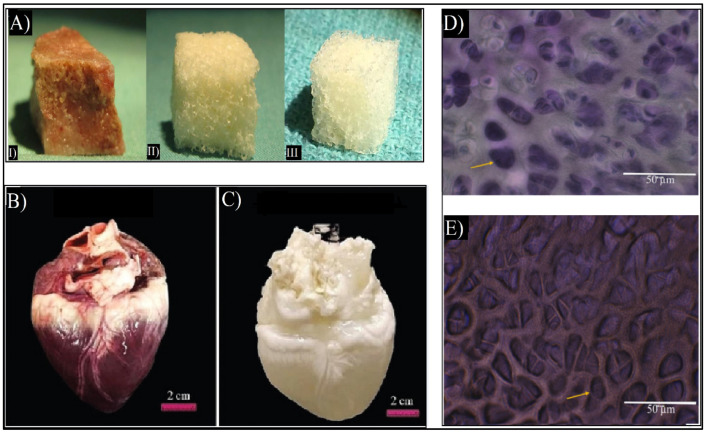
(**A**) Macroscopic images of cancellous bone (**I**), decellularized bone (**II**), and commercial demineralized bone matrix (CONFORM^®^) (**III**) [30]. (**B**) Macroscopic image of the native heart and (**C**) macroscopic image of decellularized heart. Adapted from [31]. (**D**) Flash blue staining of sectioned avian cartilage revealing the cells’ nuclei in dark blue colors of native cartilage. (**E**) Flash blue staining of decellularized avian cartilage revealed the absence of tissue cells/nuclei, showing empty pores (yellow arrows). Adapted from [32].

In order to create dECM biomaterials, decellularization protocols are being optimized for any given tissue. This is achieved by separating the cells and the nucleic acids of the original tissue and maintaining the 3D structure, chemical composition, and mechanical characteristics of the original ECMs [22]. Most tissues have been able to be decellularized, such as skin [28,29], bone [30], and heart [31], as shown in Figure 3. Other tissues, such as tendons, skeletal muscles, blood vessels, and ligaments have also been decellularized [15,26,32]. Currently, there are ECM-based implants, such as AlloDerm^®^, Oasis^®^, and Chondro-Gide^®^, as well as commercialized dECM bioinks such as Bone deCelluid™ [25,33] that are being addressed for clinical use, meaning that in vivo tests are necessary [25,34].

Typically, tissues are harvested from animals, due to their high availability. Tissue antigenicity represents the main limitation of using xenografts in clinical practice [33]. Decellularization focuses on removing cells from xenogenic tissues, reducing tissue antigenicity; however, collagens and proteoglycans from xeno-ECM have a high potential to trigger immune responses in the host due to the presence of antigens. In particular, collagen type II has been described as a cause of inflammatory response [25,33]. Furthermore, an immune rejection can be triggered by insufficient decellularization that is caused by cell residues. The success of cell removal depends on the decellularization agent that is used; therefore, there are a variety of decellularization protocols for different tissues [25,34].

Since xenoantigens have been shown to remain in acellular scaffolds, the focus has shifted to decellularization techniques that include antigen-removing steps. In particular, DNA, the alpha-Gal epitope, and major histocompatibility complex I (MHC-I) are responsible for the persistent antigenicity of decellularized xenografts that trigger hyperacute and acute rejection mechanisms and ultimately cause graft degeneration and failure [25,34]. Macrophages are responsible for the acute inflammatory reaction and the later wound healing; moreover, their behavior could be modulated with the addition of cytokines, such as IL-4, that lead to a decrease in the synthesis of inflammatory factors [35]. Although decellularized ECM is promising, the immunogenic potential has to be addressed for clinical use, meaning that in vivo tests are necessary [25,34].

The combination of 3D bioprinting and dECM bioinks is suitable for obtaining tissue-like scaffolds that are significantly similar to the original tissue [26]. The general steps for tendon decellularization and its use as a novel biomaterial in bioinks for 3D bioprinting for tissue regeneration will be reviewed.

## 2. Decellularization Process

**Figure 4:**
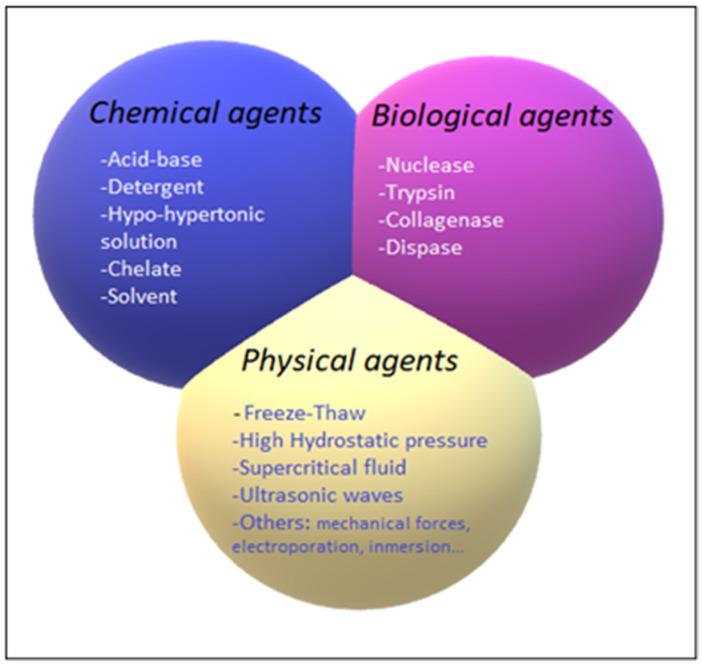
Classification of chemical (blue), biological (pink), and physical (yellow) decellularization agents.

The decellularization process is carried out by different decellularization agents, which can be classified into chemical, biological, and physical agents. The different decellularization agents are summarized in Figure 4.

### 2.1. Chemical Decellularization

The chemical agents that are used for decellularization are acid-base agents, detergents, chelating, and solvents.

Acid-base agents eliminate the cellular components, such as the nucleic acids, by dissolving them through a hydrolytic degradation process. The main problem with acid-base agents is their capability of disrupting the ECM ultrastructure, in addition to causing cytotoxicity and inducing an immune response due to the chemical residue that is caused by insufficient cleansing and incomplete decellularization. On the other hand, they have some sterilization capacity, which can save the trouble of sterilizing afterwards [17,36].

Detergents solubilize the cell membranes and the nuclear components and denature proteins. Detergents are favored among all of the chemical agents for decellularization, although the ECM can be damaged by the alteration of its protein quaternary structures. They can be classified into ionic, non-ionic, and zwitterionic detergents. Ionic detergents, such as sodium dodecyl sulfate (SDS), efficiently remove the cellular and the nuclear components, even in thicker tissues. However, SDS can thoroughly eradicate GAGs, damaging the collagen structure. Non-ionic detergents, such as Triton X-100, are weaker, however, they are better in thinner tissues [15,36,37]. Triton X-100 allows efficient cell removal while preserving the ECM [20]. Zwitterionic agents, such as 3-((3-cholamidopropyl) dimethylammonio)-1-propanesulfonate (CHAPS), have a mild effect on the tissue and often need to be combined with ionic or non-ionic detergents. Detergent-mediated decellularization can also decrease the valuable growth factors in some tissues [15,36,37]. In order to reduce the negative effects of detergents on ECM proteins, there are protective substances, such as aprotinin. It has been shown to help to maintain the properties of the tendon dECM [38]. In addition, residual chemicals may have an undesirable effect on recellularization due to their cytotoxicity in the remaining tissue [15,36,37].

Hypotonic and hypertonic solutions, such as Tris-HCl or NaCl, decellularize tissues by disrupting the DNA interactions with proteins, which can result in cell rupture by osmotic effects. Typically, it must be repeated several times. It is not as corrosive as the other methods, but it is insufficient for complete cell removal [15,36].

Chelating agents, such as ethylenediaminetetraacetic acid (EDTA) or egtazic acid (EGTA), disrupt cell adhesion by binding to metal cations. EDTA removes acid-soluble proteins; however, cellular residues may remain attached to the ECM. Thus, they are usually combined with different agents [15,36].

Solvent-like agents, such as alcohol or acetone, cause cell dehydration as a mechanism of action. They also remove lipids, in addition to having a good sterilizing capacity. However, they are tissue fixative, which can lead to protein precipitation that damages the microstructure of the ECM [15,36].

### 2.2. Biological Decellularization

Biological agents, such as nuclease, trypsin, collagenase, and dispase, can efficiently disrupt cells. Nucleases break down the DNA or the RNA, while dispase and collagenase break down fibronectin and collagen, respectively. Trypsin detaches peptide bonds, rupturing cell-matrix adhesion. However, an immune response can be triggered by the enzyme residues. Overexposure changes the ECM composition, which primarily affects the collagen, fibronectin, and GAG content. Normally, they are used in combination with chemical or physical agents as a result of them not being sufficient on their own for total cell removal. [15,36]. In order to be able to have successful tendon decellularization with fewer agents, the tendon must be finely cut. Freeze–thaw cycles, combined with nucleases, have been shown to achieve complete decellularized tendon slices that induce tendon regeneration in large tears [39]. A combination of DNAse and RNAse solutions with Triton X-100 has also been shown to successfully obtain tendon dECM [12].

### 2.3. Physical Decellularization

The most used physical agents are freeze–thaw (F–T) cycles, high hydrostatic pressure (HHP), ultrasonic waves, and the use of supercritical fluids (SF). They are responsible for lysing tissue cells by disrupting their membranes. Provided that the physical agents are used appropriately, the disruption of the ECM ultrastructure can be reduced. However, after the membrane lysis, the remaining cellular residues require further treatment in order to acquire acellular tissue [36]. Physical methods were developed in order to avoid the high toxicity of chemical agents [13]. Agitation and immersion, pressure gradient, vacuum-assisted, and perfusion flow methods are used to help to decellularize tissues [36].

F–T cycles are commonly used as a decellularization agent, causing a thermal shock that leads to the formation of intracellular ice crystals that can disrupt the cellular membrane without adding chemical agents [36]. The freezing temperatures that are needed for freeze–thawing can reach −80 °C, and the thawing process can reach temperatures of 37 °C [40]. Since it does not change the ECM mechanical properties and only causes minor changes in the tissue architecture, this method is commonly utilized in decellularization protocols [36]. Typically, the tendons are decellularized with 5–10 F–T cycles [41]. In order to effectively decellularize the tendons in a short time, a combination of F–T cycles with detergents such as SDS has been carried out [42].

HHP is considered to be a favorable method because it requires a short amount of time and has been shown to successfully decellularize tissues without altering the ECM structure. The formation of ice crystals, which is caused by water, can harm the ECM. In order to reduce the phenomenon of crystal formation, the temperature can be increased during decellularization. However, this may result in ECM vulnerability [40]. HHP can also be used with detergent solutions [43].

Using high-power ultrasonic waves can disrupt the intermolecular bonds, disrupting the cell membrane and removing its internal components, which results in breaking up the cell membrane and destroying its components. Unstable cavitations can fracture the ECM. By modifying the viscosity, the dissolved gas in the fluid, along with the temperature, can control the cavitation [43]. For example, in order to improve the decellularization process for cartilage tissue, ultrasound waves are recommended as they improve the penetration of materials into the tissue [44].

**Figure 5:**
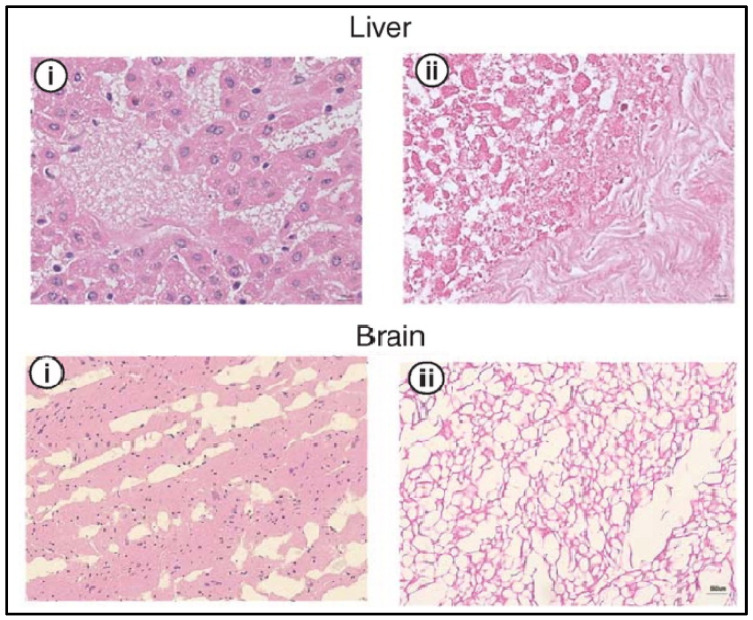
H&E analysis of the liver and brain before (**i**) and after decellularization (**ii**) with SCCO_2_. Cellular nuclei stained blue. Adapted from [37].

The use of supercritical CO_2_ (SCCO_2_) has been shown to successfully decellularize different tissues that show no toxicity and retain the ECM signals for later use as a biomaterial in bioinks, as shown in Figure 5. SCCO_2_ decellularizes by passing through the tissue once it reaches its critical parameters (7.40 MP, 31.1 °C) and transitions to the supercritical state. Nevertheless, the pressure must be thoroughly under control in order not to alter the ECM structure [36,40]. Due to its high permeability, SCCO_2_ will be rapidly removed from the tissue [40]. However, since SCCO_2_ is nonpolar, it requires an extra step in order to remove the phospholipid polar portion of the membrane [36]. This can occur with the use of ethanol, in order to remove it properly, which also allows the preservation of the tissue’s hydration [40,45]. SCCO_2_ can be combined with chemical agents in order to optimize the decellularization process with the least possible exposure and residual chemical agent on the tissue [45].

Mechanical forces can also be applied by either scraping with a scalpel or an abrasive material, along with salt solutions. The ECM structure may be compromised due to the aforementioned mechanical forces’ impact on the ECM proteins. Considering the membrane’s vulnerability to mechanical stress that is caused by excessive pressure, the required force must be accurately measured in order to prevent ECM structure damage [36,40]. Decellularization by electroporation is caused due to microsecond electrical pulses that can cause micropores to form in the cell membrane. The process above can lead to cell apoptosis due to cell loss of homeostasis. By controlling the changing pulse length and frequency, and electric field density, as well as the iteration numbers, the duration of decellularization can be manipulated. The decellularization tissue size is limited by the size of the electrodes. However, in order to prevent the immune system’s inflammatory response, decellularization must take place in vivo or with continuous vascular perfusion in order to be able to delete the cell debris [44].

**Figure 6:**
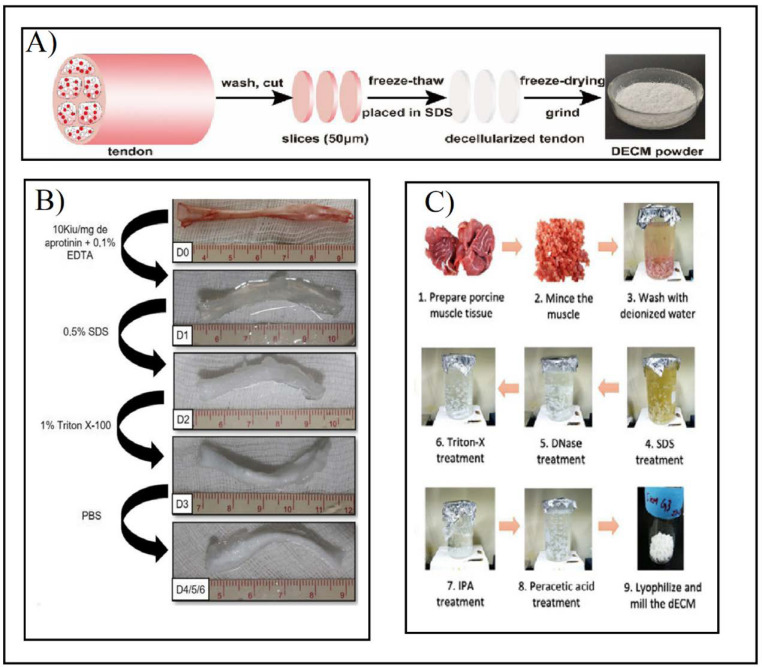
(**A**) Preparation of tendon dECM powder by combining F–T cycles and SDS after washing, cutting, and slicing the tendon. Adapted from [42]. (**B**) Multi-stage tendon decellularization protocol. Aprotinin and EDTA were added to fresh tendons (D0). Then 0.5% SDS (D1) and 1% Triton X-100 (D2) were added in consecutive steps. Finally, it was washed with PBS (D3) to obtain the final decellularized tendon (D4/5/6). Adapted from [38]. (**C**) Minced and washed muscle decellularization under immersion and agitation combined with SDS (1–4), DNAse treatment (5), and Triton X-100 (6) agents. After the decellularization process, the muscle was immersed in antibiotic solution (7), and then sterilized with peracetic acid (8). Ultimately it was lyophilized and milled (9). Adapted from [45].

Agitation and immersion eventually may lead to cell apoptosis and are commonly used to assist the agent in reaching the tissue cells [36]. Various factors, such as tissue thickness, the detergent that is used, and the intensity of agitation, affect the duration of the decellularization [17]. There are various ways to induce the agitation process, the most common of which are an ultrasound bath, a rotating chamber, or a magnetic plate. The agitation that is caused by severe stirring or ultrasound can damage the ECM [40,45]. It can shorten the decellularization time, as the organ can be minced in order to increase the surface area, as seen in Figure 6C [45]. The pressure gradient can help the chemical decellularization agents to penetrate the tissue more efficiently but can destroy the ECM structure. Although it decreases the time that is needed to complete the penetration, it requires fewer abrasive agents to obtain a similar level of decellularization. Vacuum-assisted methods can expedite and enhance the chemical agents’ efficiency in the tissue. Another application of the vacuum methodology is the removal of the detergents from decellularized tissues. Perfusion flow decellularization is applied by separating the organ from the blood vessels and injecting the chemical agents into its vascular system. By doing this, the removal of the cells and their components can be facilitated. One essential step in this process is that the flow level must be controlled. This is due to the damage capability of the pressure that is used on the capillaries [40]; the ECM structure may be disrupted due to the perfusion pressure [40,45]. With this method, the entire structure of the organ is preserved, but it requires specialized bioreactors [45]. This process may not be feasible in all organs due to some organs lacking the required vessel net in order to allow perfusion and to achieve decellularization; in that case, the organ is submerged in decellularization agents with agitation [15].

The decellularization process is mainly controlled by various parameters, such as the type, the concentration, the exposure time, the temperature, and the pH of the agent [45]. Although the physical methods cause the least damage to the tissue structure, they fail to eliminate the genetic material, thus causing significant immune responses. On the other hand, chemical agents, such as detergents at low concentrations or enzymatic agents alone, cannot eliminate the cell debris [20,40]; hence, additional processes are needed in order to achieve it. In order to help to make this feasible, multi-stage protocols that include physical, chemical, and biological methods need to be used, as shown in Figure 6 [26,40].

## 3. Analysis of the dECM

The lack of universal standards for evaluating the completion of the decellularization process can be a problem. The primary evaluated variable is the DNA content that is present in the decellularized product. In order to confirm the absence of cellular components in the decellularized ECM, DNA quantification and hematoxylin and eosin (H&E) staining must be performed. The decellularization efficiency is determined by measuring the abundance of nucleic acids in the cells by utilizing DAPI staining or H&E. There are two parameters for a decellularization process to be considered successful, as follows: (a) containing a concentration lower than 50 ng of double-stranded DNA (dsDNA)/mg dry dECM, or (b) a length of no more than 200 base pairs without noticeable nuclear material. Regardless, these conditions are not unanimously stipulated by any entity, but they are respected and abided by. Other methods exist to characterize decellularization, such as tissue color inspection, histological assays, and measuring detergent residues. ECM components, such as collagen, elastin, GAG, and content, as well as xenogenic factors such as gal-epitope, can be measured [45,46]. Proteomic analysis is also helpful because it facilitates both compositional and quantitative data [46].

The literature mainly focuses on the biochemical properties of the dECM [47]. As mentioned previously, decellularization affects the composition and the structure of molecules and proteins. This impact can be measured qualitatively and quantitatively by the use of spectroscopy methods or ELISA, SDS page analysis, and western blot analysis. The collagen content is measured by using hydroxyproline assay, while dimethylene blue-based assays quantify GAGs. HPLC-based assays can measure the residual chemicals in the dECM [27,48,49].

The physical properties should also be evaluated since they may have significant roles in biological circumstances. The primary physical properties that are evaluated are the fiber organization and the scaffold structure, which is mainly carried out through SEM analysis; however, analysis with X-ray, CT-scan, Cryo-EM can also be conducted. Information on different material properties, such as the architecture, the surface density, the porosity, the particle size, and the surface feature profile of the fiber diameter can be obtained with the named techniques [27,47].

**Figure 7:**
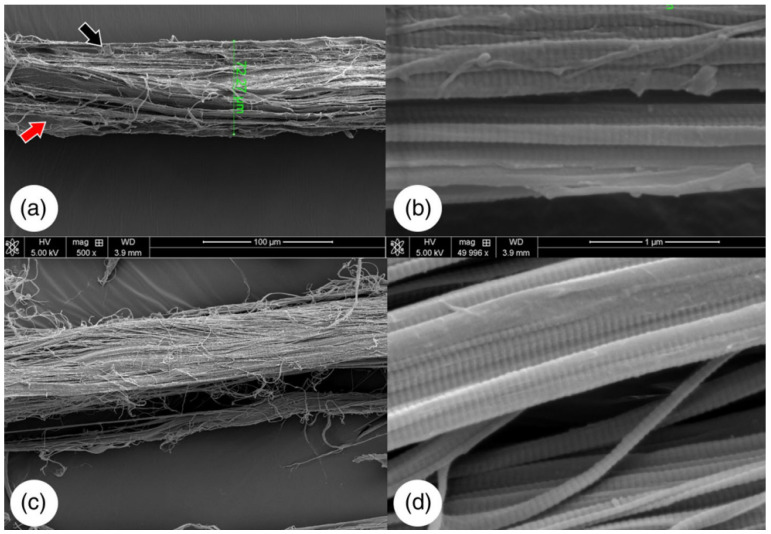
SEM images of native bovine tendon (**a,b**). The red and black arrows are pointing at cellular debris. Decellularized bovine tendon (**c**,**d**). Adapted from [50].

The properties of the dECM materials, such as the elasticity, the bendability, and the tensile properties, can be majorly affected by variations in fiber thickness [47]. After decellularization, the collagen fibers can be maintained, as shown in Figure 7.

The study of properties such as young’s modulus, storage modulus, tensile strength and elasticity through rheology, uniaxial tensile and compressive testing, DMA, FTIR, and AFM can be performed both before and after decellularization in order to identify any harmful effects on the tissue [47]. These physical properties are very compelling in hard tissues or tissues that have to bear great tensile strength, such as articular cartilage [8]. Other protocols include the characterization of viscosity, density, surface wettability, degradation, and thermal properties [47].

The proteins are essential components of the ECM, and they determine its biomimetic characteristics. Hence, proteomic characterization and deep analysis of the dECM are vital. Numerous proteins were decoded in multiple tissues for characterizing the gene expressions that are associated with the maturation and the differentiation of reseeded cells in the dECM [48]. LC-MS/MS is the most widely accepted technique, while other approaches include mRNA microarrays or quantitative or real-time PCR [12,48].

Some essential genes for characterization include RUNX2, COL1A1, COL2A1, COL3A1 or SCX, ACAN, SOX 9, TNC, TNMD, and LAMA1 [12]. Genes such as COL2A1, ACAN, or SOX 9 are chondrogenic differentiation markers. SOX-9 plays a vital role in activating the expression of genes that are associated with cartilage ECM, such as ACAN and COL II, while suppressing fibrotic-related gene expression, such as COL-I. RUNX2 is a transcription gene for osteoblast differentiation. One of the transcription factors that is found explicitly in tendon precursor and mature tendon cells is SCX, thus becoming a marker for tendon and ligament tissues. COL3A1, TNC, and TNMD are genes that are related to tendon ECM (Table 1), while LAMA1 is related to signaling, cell adhesion, migration, and differentiation [11,12,42]. Collagen type 1 is expressed by mature tenocytes. The detection of additional ECM proteins can help to detect tissue-specific cues, such as prolargin and fibromodulin, for tendons [42,48].

Proteomics can also be used to detect proteins in the dECM that can be used as a successful decellularization biomarker. These proteins are summarized in Table 2 [51].

Furthermore, the growth factors that are necessary for cell functions such as proliferation, migration, and differentiation, such as insulin-like growth factor 1 (IGF-1), vascular endothelial growth factor (VEGF), transforming growth factor beta (TGF-β), and basic fibroblast growth factor (bFGF), can be quantified. The bFGF promotes tenogenesis and participates in repairing the tendon, as it helps in the synthesis of GAG and collagen and serves to preserve the undifferentiated state of its stem cells. IGF-I facilitates healing in tissues such as tendons and cartilages, plays an essential role in various physiological processes, and promotes the synthesis of fibrous tissues with increased crosslinking. VEGF is an essential angiogenic factor that has the ability to enhance endothelial cell proliferation and vascular permeability. TGF-β1 is crucial for cell growth and is remarkably ample in cartilage tissue. It promotes differentiation and proliferation [11,12]. TGF-β1 can achieve tissue adhesion by initiating fibrotic modification in the process of tendon reparation [52].

## 4. dECM Sterilization and Preservation

Eventually, the dECM needs to be in a sterile state. Protein denaturation may be inevitably caused by applying conventional sterilization techniques, such as dry heating, pressurizing, and chemical use [48]. In order to achieve a sterilized dECM, the following conditions must be met:

(a) Effectively remove microorganisms;

(b) Make sure that the sterilized materials are non-toxic;

(c) Maintenance of the chemical and physical characteristics of the dECM [53].

**Figure 8:**
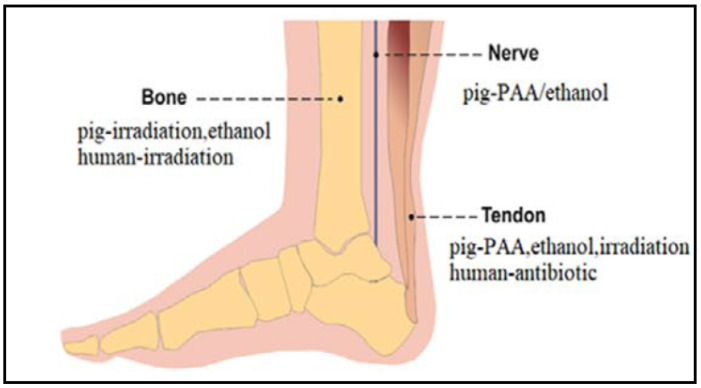
Recommended sterilization methods of bone, nerve, and tendon pig-derived dECMs. Adapted from [54].

To sterilize the dECM scaffolds, ethylene oxide and gamma irradiation are among the most widely used methods. However, gamma irradiation can change the dECM characteristics, such as the chemical and the physical properties. It damages the structure and the mechanical properties of the dECM. On the other hand, ethylene oxide causes protein damage and is considered to be cytotoxic. Depending on the tissue of the dECM, different sterilization methods are recommended, as shown in Figure 8 [14,54]. Hence, the sterilizing agent must ideally be safe and straightforward to use, along with having sufficient sterilization properties [48]. Other sterilization and disinfection methods, such as peroxide, alcohol, ultraviolet ray, supercritical carbon dioxide, and antibiotics, have been applied to dECM in order to avoid infections [53]. For tendon dECM, the most common sterilization methods are peracetic acid (PAA), ethanol, gamma irradiation, and antibiotics [54,55].

Preservation methods such as cryopreservation and lyophilization are commonly used; however, the biological composition, the scaffold aspect, and the mechanics can be heavily altered. For cryopreservation, agents such as DMSO and glycerol are used to preserve the properties of the tissue. However, it has been shown to change the morphology, the mechanical properties, and the biomechanical behavior of the scaffolds. The preservation agents can be toxic as well [54].

Lyophilization has become the best alternative since it is non-toxic, cheaper, and easier to use. In addition, after using the lyophilizer, it is feasible to turn the dECM into powder for its subsequent digestion and bioink formulation [54].

## 5. Decellularization Protocols for Tendons

As mentioned previously, depending on the selected tissue, decellularization protocols can differ. In other MSK tissues, such as cartilage [56,57,58,59,60,61] and ligaments [43,62,63,64,65,66,67], similar protocols can be used with different outcomes. However, the results have been shown to change when applying the decellularization protocol with a different harvest site of the same tissue [56].

Different decellularization protocols for tendon tissue are summarized in Table 3. As generally agreed, the dECMs have less than 50 ng/mg per dry weight after the decellularization process. In general, dECM are successfully obtained with different influences on the final dECM composition. The treatment with 1% SDS preserved the mechanical and structural properties of the tendon [55]. Higher concentrations, or its combination with Triton X-100, lead to a worse structure and GAG content [68]. The use of biological agents has been shown to preserve the collagen content but with the loss of the mechanical properties of the scaffold [50]. Jones et al. developed a protocol with a drastic depletion of GAG content but maintained mechanical properties. They were able to remove collagen II, decreasing further in vivo inflammatory response, but not the alpha-Gal epitope completely [69]. The combination of EDTA and SDS has been shown to remove the MHC-I from the tendon dECM [55].

Changes in the dECM and residual decellularization agents affect the posterior recellularization of tendons. The dECM that are treated with SDS are less receptive to cellular growth than the other detergents [55].

Although the in vitro studies show promising results, there are only two studies that have performed in vivo testing, both of which were subcutaneous nude mice tests, where the real conditions for tendon repair were not met [68,69].

## 6. Tendon-Derived dECM Bioinks

Due to its tissue-specific complex composition, dECM is considered to be a promising material for preparing bioink [15,46,72]. This stems from its capacity to provide crucial biochemical elements and to preserve mechanical and structural features that are key to cell viability [47]. Four characteristics need to be evaluated before using dECM as bioink, which are as follows: the cell compatibility, the mechanical properties, the printability, and the remodeling capacity [46]. In order to further develop the medical usability of the dECM, the material can be polymerized into a soluble form that can be made into a gel. This can help to prevent the drawbacks of using the dECM as a biological sheet or organ, such as donor shortage and shape limitations [46,48].

**Figure 9:**
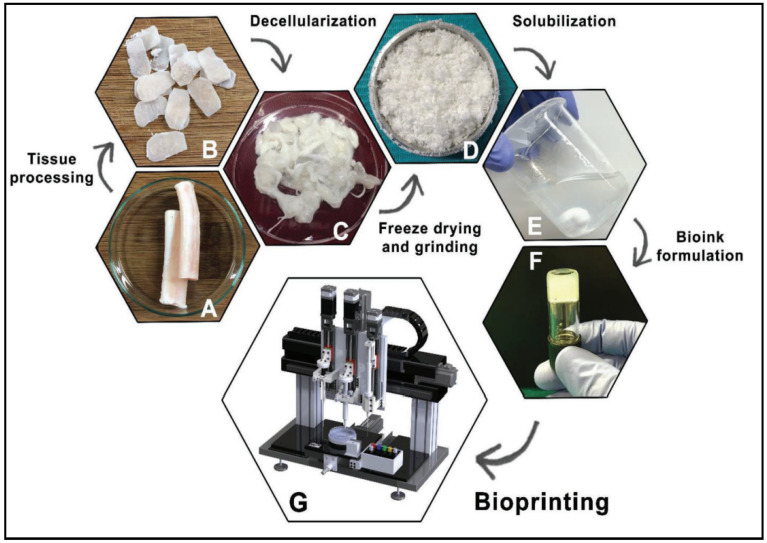
General steps to obtain a dECM bioink. Native tissue (**A**), Processed tissue (**B**), Decellularized tissue (**C**), Freeze-drying and grinding (**D**), Solubilization (**E**), Bioink formulation (**F**), Bioprinting (**G**). Reproduced with permission [73].

Upon obtaining the soluble form, after the completion of the decellularization protocol and lyophilization of the dECM, the dECM is powdered. Afterwards, it will be digested by pepsin or urea in order to obtain a viscous solution. Urea-digested dECM has shown to be better since it retains more growth factors than pepsin-digested dECM. This widely used method, known as ECM digestion, allows the dECM to self-assemble into a gel for 3D bioprinting [12,48]. These general steps are summarized in Figure 9.

The dECM proteins such as fibronectin and collagen provide the gelation process, with responsiveness to temperature alterations, and initiate the construction of a crosslinked network, provided that it is incubated at 37 °C, which results in a crosslinking of the bioink. Such powders have gained popularity for their high efficiency, accessibility, and preserving signaling molecules. Those decellularized tissue particles are used to enhance biomaterials with low biocompatibility in 3D bioprinting; dECM powders are known to possess some distinctive features, such as lower invasive intervention, accessibility, bioactivity, and significant efficiency [26,74].

There are various studies where dECM bioinks for tendons are developed [54], which are summarized in Table 4. Although dECM-based bioinks have poor mechanical properties, there are studies where they only use the dECM for the bioink [73,75,76]. In order to improve the properties of the scaffolds, the bioinks combine different biomaterials, or/and they may need an additional supporting structure in order to be able to achieve increasingly intricate 3D models [74,77].

The study of tendon regeneration using dECM bioinks has been increasing with the development of new dECM and bioprinting techniques [46,73,78,79]. The first steps include the optimization of parameters for the dECM preparation, such as the reagents that are used or the digestion time, in order to improve the 3D bioprinting. Fengyuan et al. demonstrated that different acidic solutions for the solubilization of tendon dECM directly impact the 3D bioprinting step. A porcine-derived decellularized bioink, which was solubilized with 0.1 M hydrochloric acid, provided a much softer tendon-derived dECM hydrogel with a storage modulus of less than 100 Pa, facilitating the spreading and the proliferation of the cells that were encapsulated in it, and showed better tendon-inducing ability. However, it was more unstable as it could shrink with time. Solubilizing with 0.5 M of acetic acid leads to much lower cellular viability rates due to the hyperosmotic state of the bioink. A postprocessing step, such as dilution or dialysis, could be necessary in order to address the osmotic pressure before mixing the dECM ink with cells. Different acidic solutions lead to a different gene expression profile and changes in the rheological properties [78].

**Figure 10:**
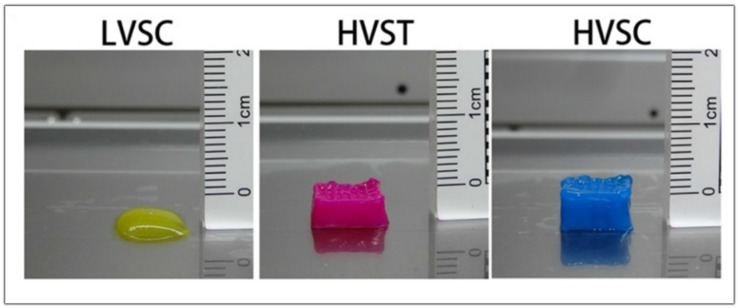
Images of the scaffold obtained with low viscosity cylindrical tip (LVSC), high viscosity tapered tip (HVST), and high viscosity cylindrical tip (HVSC). Adapted from [79].

In another study, Fengyuan et al. compared the digestion time of the dECM and the printability of the bioink. When only the dECM is used in the hydrogel, better printability is obtained when the digestion time is lower as the viscosity gradually decreases with the increasing digestion time. The low viscosity of the bioink and the slow gelation properties could make printing difficult without the addition of supporting materials such as gelatin methacrylate or hyaluronic acid. With three hours of digestion, they obtained a high viscosity bioink that showed better cell viability when it was bioprinted with a tapered tip. When using a tapered tip, less pressure is applied when bioprinting than with a cylindrical needle (30 kPa vs. 140 kPa, respectively). The high shear stress can lead the cells to apoptosis, reducing their viability. When bioprinting a high viscosity bioink, the resulting scaffold is more precise and has more layers than a low viscosity bioink, as shown in Figure 10. However, in order to measure the rheological characteristics and analyze the self-assembly, further studies are to be conducted in order to facilitate their future application in 3D Bioprinting [79].

**Figure 11:**
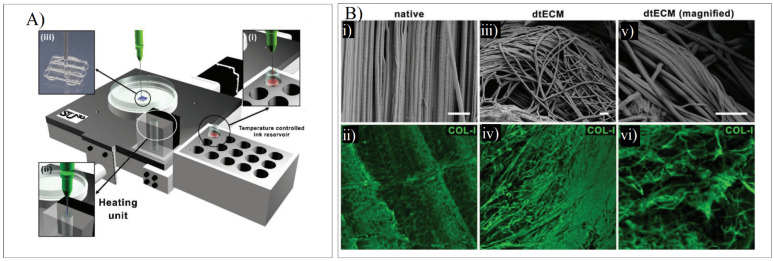
(**A**) Schematic representation of the printing process where the bioink from a reservoir (**i**) is taken to the heating unit (**ii**) to bioprint the scaffold (**iii**). (**B**) SEM and COL-I immunostaining in native tendon (**i**,**ii**) and dECM tendon (**iii**–**vi**). Adapted from [73].

When developing a dECM bioink for tendon regeneration, Toprakhisar et al. obtained a hydrogel from decellularized bovine Achilles tendon with no need for an extra crosslinker or a reinforcement structure by modifying the developed dECM hydrogels’ gelation kinetics. This was carried out by using an aspiration–extrusion bioprinting protocol, as shown in Figure 11A. Firstly, pre-gel solutions’ aspiration is followed by in situ gelation before extrusion by heating the pre-gel solution at 37 °C [73].

The SEM imaging and the Col-I immunostaining demonstrated that collagen fibers were piled together in the same direction as the native tissue. The collagen-specific pattern was maintained despite the deformation that was present in the decellularized samples. The fibers were unharmed, showing arbitrarily aligned collagen I fibers, as shown in Figure 11B [73].

The viability of the 3T3 fibroblasts was higher than 75%, and the average cell circularity remarkably declined after the third day, leading to lineage morphology. The maximum compressive stresses and the compressive moduli increased in tandem with increasing dECM hydrogel concentration. This is directly associated with a growth in the density of the collagen fibers in the hydrogel. The longer the digestion times were, the poorer the network mechanical strength. Since the dECM elastic moduli are still considerably lower than the bovine Achilles tendon, the mechanical characterizations can be used as an indicator of the formation of a stable hydrogel [73].

For the regeneration of the tendon–bone interface (TBI), which is present in rotator cuff (RC) injuries, a 3D bioprinted scaffold mimicking the multi-tissue characteristics of TBI was developed [80]. The RC is composed of four tendinous entheses consisting of the following three distinct, yet continuous, tissue layers: the tendon, the fibrocartilage, and the bone [6]. For that purpose, different TBI-specific bioinks were made. The Achilles’ tendons were decellularized and pepsin-digested to a final concentration of 20 mg/mL, obtaining the tendon-derived extracellular matrix (TdECM) bioink. The other bioinks were composed of bone-derived extracellular matrix (BdECM) and polyurethane/poly-caprolactone (PU/PCL) [80].

**Figure 12:**
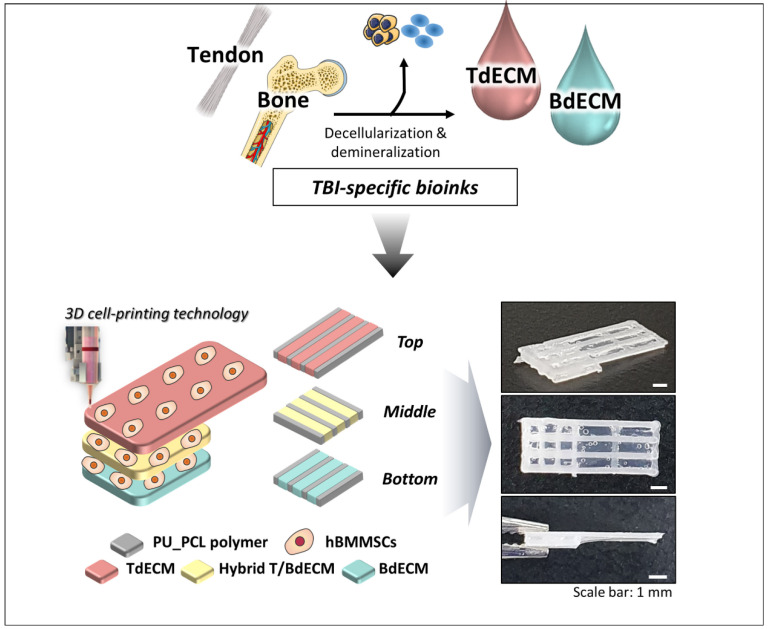
Illustration of how tendon and bone dECM bioinks with hBMSCs in different layers create unique 3D bioprinted scaffolds for RC regeneration. Adapted from [80].

As seen in Figure 12, alternate strands of the PU/PCL and the dECM bioinks were bioprinted with hBMMSCs. The PU/PCL ink is necessary to provide structural support. For the bottom layer, BdECM was used to promote osteogenesis. Meanwhile, the middle layer is composed of a hybrid BdECM and TdECM bioink that seeks to promote fibrochondrogenesis. The top layer consists of TdECM bioink, corresponding to the tendon region. After bioprinting, the scaffolds were incubated at 37 °C in order to induce the bioink gelation [80].

**Figure 13:**
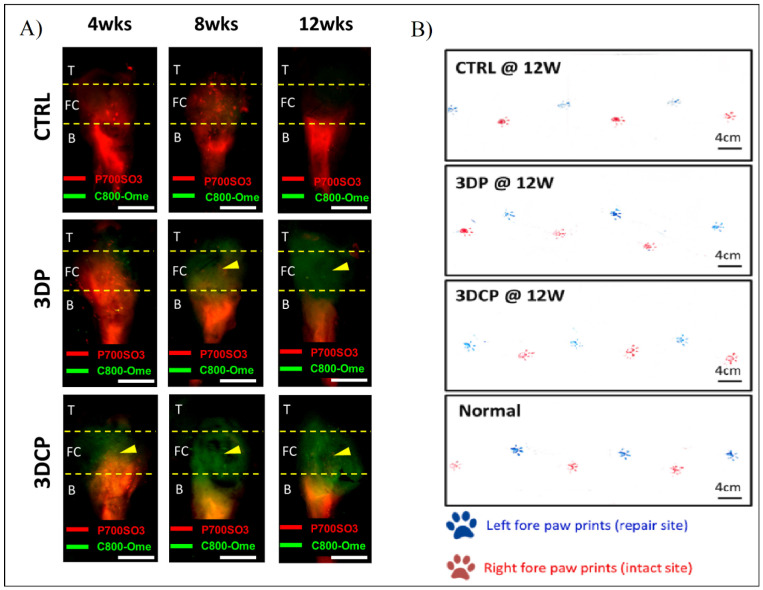
(**A**) Fluorescent evaluation of dECM tendon–bone scaffold for RC regeneration in control (CTRL), decellularized scaffold (3DP), and recellularized scaffold (3DCP) after 4, 8, and 12 weeks post-operation. (**B**) Gait analysis after 12 weeks post-operation of the left paw (blue, repair site) and right paw (red, intact) of CTRL, 3DP, and 3DCP after 12 weeks, and normal steps. Adapted from [80].

In order to detect the in vivo regeneration and differentiation, they designed a non-invasive dual fluorescent system to evaluate the formation of the bone, the fibrocartilage, and the tendon regions using labeling fluorophores in rat RC models. This study, along with others such as gait analysis and histological and mechanical evaluation (Figure 13), show better results with the multiphasic bioink containing hBMMSCs than the control and the same bioink without cells [80].

**Figure 14:**
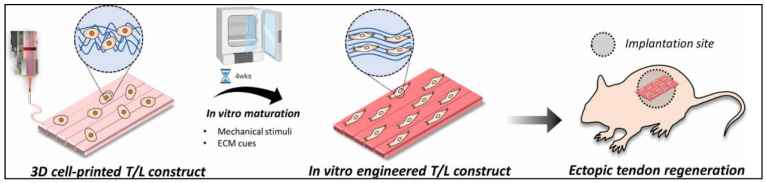
Illustration of how the 3D bioprinted scaffold underwent in vitro maturation for 4 weeks in order to obtain a more aligned and differentiated scaffold. Lastly, the study of ectopic tendon regeneration was performed. Reproduced with permission [81].

In another study, Chae S. et al. developed a scaffold for the tendon–ligament interface using decellularized Achilles porcine tendon. The bioink was only composed of TdECM and hBMMSCs. In order to improve the printability of the bioink, they first created a framework with PCL and then bioprinted it in a supporting bath of gelatin granule. This helps to maintain the shape fidelity, as it entraps the bioink. After the scaffold’s gelation, the support bath was cleared. After that, they placed the scaffolds for in vitro preconditioning. They were under mechanical stimuli for four weeks. For the in vivo studies, only the ectopic tendon regeneration studies were performed. The general steps of the strategy for T/L scaffolds are shown in Figure 14 [81].

**Figure 15:**
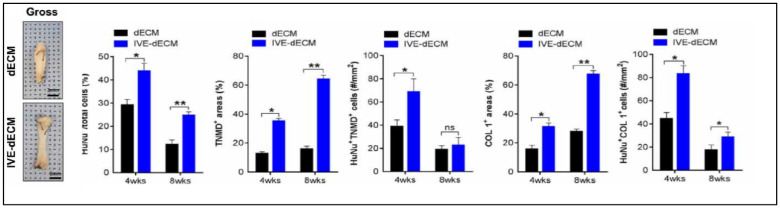
Gross images of dECM and in vitro stimulated dECM (IVE-dECM) tendon scaffolds. Characterization of cell area, TNMD, and COL-1 expression of dECM (black) and IVE-dECM (blue) tendon scaffolds after 4 and 8 weeks post-surgery is shown. (* *p* < 0.05 and ** *p* < 0.01). Adapted from [81].

During in vitro maturation, the ECM signals that were present in the scaffold facilitated long-term in vitro culture with high cell viability and enhanced tenogenesis. They also obtained improved cellular anisotropy, thus creating well-aligned structures with better mechanical strength, as shown in Figure 15 [81].

## 7. Conclusions

The tendons are a part of the musculoskeletal system and they have many vital functions, especially movement-related. After an injury, their complete regeneration cannot be carried out without assistance, due to their limited regeneration capabilities. Novel techniques, such as tissue decellularization, offer a new approach to mimicking the native ECM complex microenvironment. The objective of tissue decellularization is to maintain the native ECM structure and biological cues while removing cells and cell debris. Since dECM are mainly harvested from animals, the absence of native cells minimizes the risk of eliciting an immune response in the patient.

Depending on the tissue, different decellularization protocols are developed. For example, for tendons, the most used agents are detergents, such as SDS or Triton X-100, combined with biological agents, hypotonic and hypertonic solutions, and freeze–thaw cycles. Although there is no agreement on which method is better, detergents are preferred. Other methods can include the addition of HHP, supercritical fluids, and perfusion in order to improve decellularization.

Present studies demonstrate that the use of dECM as an active component of 3D bioprinting bioink is promising. One of the advantages is the ability to express tissue-specific cues leading to MSCs differentiation towards the dECM tissue source. However, the lack of standardized decellularization protocols for any given tissue makes reproducibility hard. Another limitation is the lack of standardized characterization of the dECM bioinks, which leaves it up to the researchers to decide on a specific characterization. However, parameters such as DNA, collagen, and GAG content are usually unanimously accepted. The characterization should be performed pre-and post-decellularization in order to evaluate the repercussions on the dECM characteristics.

The bioinks that are made only with dECM have poor mechanical properties, structural integrity, and slow gelation properties. Therefore, the common strategy is to combine the bioink with different biomaterials, such as hyaluronic acid or additional crosslinking agents. Lastly, printing parameters need to be established.

## Figures and Tables

**Table 1:** Specific tissue genes to evaluate for tendon regeneration.

Specific Tissue Genes	
Chondrogenic differentiation markers	COL2A1, ACAN, SOX-9
Tenogenic differentiation markers	SCX, TNMD, TNC
Cartilage marker	GAPDH, HPRT1
Tendon marker	COL1A1, COL3A1

**Table 2:** Different decellularization biomarkers in the ECM.

Decellularization Biomarkers	
Nuclear	APTX, UIMC1, DMRT1, H3F3A
Golgi-specific	B3GALNT1, MAN1A1, FUT2
Mitochondrial	ACO2, AKAP10, GOT2
Non-Collagenous extracellular matrix proteins	ACAN

**Table 3:** Tendon decellularization protocols.

Source	Decellularization Method	Characterization	In Vivo	Observations	Ref.
Porcine super Flexor Tendon	Freeze–thaw cycle Hypotonic buffer 0.1% SDS Nuclease solution Hypertonic buffer	Good results in the uniaxial tensile testing	No	Optimized an irradiation of 25 kGy for sterilization	[70]
Porcine super flexor tendon	Freeze–thaw cycle Hypotonic buffer Proteinase inhibitor SDS Nuclease Hypertonic buffer	Presence of Col-I, III, and tenascin C, but no Col II Significant depletion of GAG content, <1% of dry weight	Subcutaneous mice model, no inflammatory or immune response was observed	Maintained strength between native and dECM groups (52.5 mPA vs. 62 mPA, respectively) Presence of a Gal epitope after decellularization	[69]
Aquiles Porcine tendon	1% SDS 0.2% sodium azide 5 mM EDTA Protease cocktail 0.05% trypsin 0.053 mM EDTA 3%Triton X-100	Important structure and GAG loss	On subcutaneous nude mice test, no inflammation was detected	Compared macroscopic appearance Lack of quantitative comparison Animal conditions after surgery does not represent real conditions	[68]
Extensor and flexor bovine tendon	Lyophilization Hypotonic buffer 0.05%trypsin Hanks Buffer Protease inhibitor Antibiotic solution	Conservation of collagen content and structure	No	Lower mechanical properties than control	[50]
SDF horse tendon	Freeze–thaw Hypotonic solution (Tris 1 M) 1%Triton X-100	Evaluation of tenogenic extracellular proteins (collagen 1A2, collagen 3A1, decorin, and tenascin c)	No	Gene expression evaluation	[71]

**Table 4:** Optimized protocols for tendon dECM bioinks for 3D bioprinting.

Tendon Source	Decellularization Protocol	Sterilization	Conservation	Digestion Method	3D Bioprinting Technique	Bioink Composition	Reference
Porcine	1: 0.25% Trypsin-EDTA at 4 °C for 48 h 2: 0.5% SDS + 0.5% Triton X-100 for 48 h	0.1% PAA + 4% ethanol for 4 h	Freeze-dried	3 mg/mL pepsin in 0.1 M HCL	Extrusion	2.5% dECM + SD-BMSCs	[78]
Porcine	1: 0.25% Trypsin-EDTA overnight 2: 2% SDS for 96 h	100% ethanol for 30 min + 70% ethanol at 4 °C overnight	Freeze-died	3 mg/mL pepsin in 0.1 M HCL	Extrusion	3% dECM + SD-BMSCs	[79]
Bovine Achilles tendon	1: 5 F–T cycles with alternate hypo/hypertonic solution 2: 0.05%Trypsin EDTA for 30 min 3: 2%SDS for 4 days	Antibiotic solution	Freeze-dried	1 mg/mL pepsin in 0.01 M HCL	Piston-driven aspiration-extrusion	10 mg/mL dECM + NIH 3T3 cell	[73]
Porcine Achilles tendon	1: 5 F–T cycles 2: 0.5% Trypsin + EDTA at 37 °C for 6 h 3: 2% Triton X-100 at 21 °C for 3 days 4.50 U/mL DNAse at 37 °C for 2 days	0.1% PAA in 4% ethanol for 3 h	Freeze-dried	10% pepsin in 0.5 M acetic acid	Extrusion	20 mg/mL TdECM + 40 mg/mL BdECM + hBMSCs	[80]
Porcine Achilles tendon	1: 6 F–T cycles 2: 0.5% Trypsin + EDTA at 37 °C for 6 h 3: 2% Triton X-100 at 21 °C for 3 days 4.50 U/mL DNAse at 37 °C for 2 days	0.1% PAA in 4% ethanol for 3 h	Freeze-dried	1 mg/mL in 0.5 M glacial acetic acid	Extrusion	2% TdECM + 2% LidECM + hBMMSCs	[81]

## Data Availability

Not applicable.
